# *Akkermansia muciniphila* exerts immunomodulatory and anti-inflammatory effects on gliadin-stimulated THP-1 derived macrophages

**DOI:** 10.1038/s41598-023-30266-y

**Published:** 2023-02-24

**Authors:** Sara Molaaghaee-Rouzbahani, Nastaran Asri, Anna Sapone, Kaveh Baghaei, Abbas Yadegar, Davar Amani, Mohammad Rostami-Nejad

**Affiliations:** 1 Department of Immunology, School of Medicine, Shahid Beheshti University of Medical Sciences, Tehran, Iran; 2 Gastroenterology and Liver Diseases Research Center, Research Institute for Gastroenterology and Liver Diseases, Shahid Beheshti University of Medical Sciences, Tehran, Iran; 3 Center for Celiac Research and Treatment, Massachusetts General Hospital, Boston, MA 02114 USA; 4 Foodborne and Waterborne Diseases Research Center, Research Institute for Gastroenterology and Liver Diseases, Shahid Beheshti University of Medical Sciences, Tehran, Iran

**Keywords:** Immunology, Molecular biology

## Abstract

Macrophages (MQs) pro-inflammatory phenotype is triggered by gliadin peptides. *Akkermansia muciniphila* (*A. muciniphila*) showed to enhance the anti-inflammatory phenotype of MQs. This study aimed to investigate the anti-inflammatory effects of *A. muciniphila*, on gliadin stimulated THP-1 derived macrophages. THP-1 cell line monocytes were differentiated into MQs by phorbol 12-myristate 13-acetate (PMA). MQs were treated with *A. muciniphila* before and after stimulation with gliadin (pre- and post-treat). CD11b, as a marker of macrophage differentiation, and CD206 and CD80, as M1 and M2 markers, were evaluated by flow cytometry technique. The mRNA expression of TGF-β, IL-6, and IL-10 and protein levels of IL-10 and TNF-α were measured by RT-PCR and ELISA techniques, respectively. Results show an increased percentage of M1 phenotype and release of proinflammatory cytokines (like TNF-α and IL-6) by macrophages upon incubation with gliadin. Pre- and post-treatment of gliadin-stimulated macrophages with *A. muciniphila* induced M2 phenotype associated with decreased proinflammatory (IL-6, TNF-α) and increased anti-inflammatory (IL-10, TGF-β) cytokines expression relative to the group that was treated with gliadin alone. This study suggests the potential beneficial effect of *A. muciniphila* on gliadin-stimulated MQs and the importance of future studies focusing on their exact mechanism of action on these cells.

## Introduction

Macrophages (MQs) are mononuclear phagocytes with important roles in innate and adaptive immune responses. These cells are present in almost all body tissues and because of their ability to recognize pathogen-associated molecular patterns (PAMPs) and damage-associated molecular patterns (DAMPs), they can efficiently and rapidly detect pathogens and tissue destructions^1,2^. MQs can polarize to M1 or M2 functional phenotypes in response to their tissue microenvironment^3^. While the M1 phenotype of macrophages expresses numerous pro-inflammatory mediators, in particular tumor necrosis factor (TNF-α), interleukin (IL)-1, IL-6, reactive nitrogen and oxygen intermediates, the M2 phenotype of them expresses molecules with immunoregulatory functions including Arginase1, chitinase 3-like 3, resistin-like-α (also known as Fizz1), IL-10 and Mrc1 (also known as CD206)^4^. M1 and M2 phenotypes of macrophages can be converted into each other in response to microenvironmental stimuli^5^.

There are numerous MQs in the lamina propria of the small intestine, with a dual role of maintaining tissue homeostasis and participating in the development of intestinal inflammation. According to the reports, gliadin peptides, which trigger immune reactions in celiac disease (CeD), induce the activation of macrophages toward a pro-inflammatory M1 phenotype^6–10^. In vitro studies with murine MQ and human monocytic cell lines have shown that gliadin triggers the production of TNF-α, IL-8, RANTES, IL-1β and significantly increases nitric oxide (NO) upon activation of toll-like receptors 2 and 4 (TLR2/TLR4)^9^.

Celiac disease is an immune-mediated systemic disorder characterized by enteropathy of the small intestine and nutritional deficiencies affecting approximately 1% of the general population varying by continent^11^. The only therapeutic strategy for this disorder is strict adherence to a gluten free diet, which is difficult to maintain^12^. Therefore, researchers planned various in vitro and in vivo studies with the specific aim of finding a new therapeutic approach for this group of patients. Nowadays, gut dysbiosis (which is commonly observed in CeD patients) and its effect on immune system responses have become a subject of interest for finding an alternative therapeutic approach^13^.

Gut bacteria *Akkermansia muciniphila* is an anaerobic gram-negative bacterium that helps maintain homeostasis and barrier integrity in the gastrointestinal tract^14,15^. In addition, several studies have shown the anti-inflammatory effects of *A. muciniphila*^16^. These genera are known to break down mucus and produce short-chain fatty acids (SCFAs), which in turn support enterocyte health and inhibit intestinal inflammation^17^.

In the present study, gliadin stimulated THP-1 derived macrophage was treated with *A. muciniphila.* The idea of this treatment was to evaluate *A. muciniphila* enhanced phenotype switching of gliadin triggered M1 MQs to an anti-inflammatory phenotype. The results may give rise to more detailed studies on this gut bacterium's beneficial effects on CeD patients' treatment.

## Results

### Differentiation of THP-1 monocytes to macrophages

**Figure 1:**
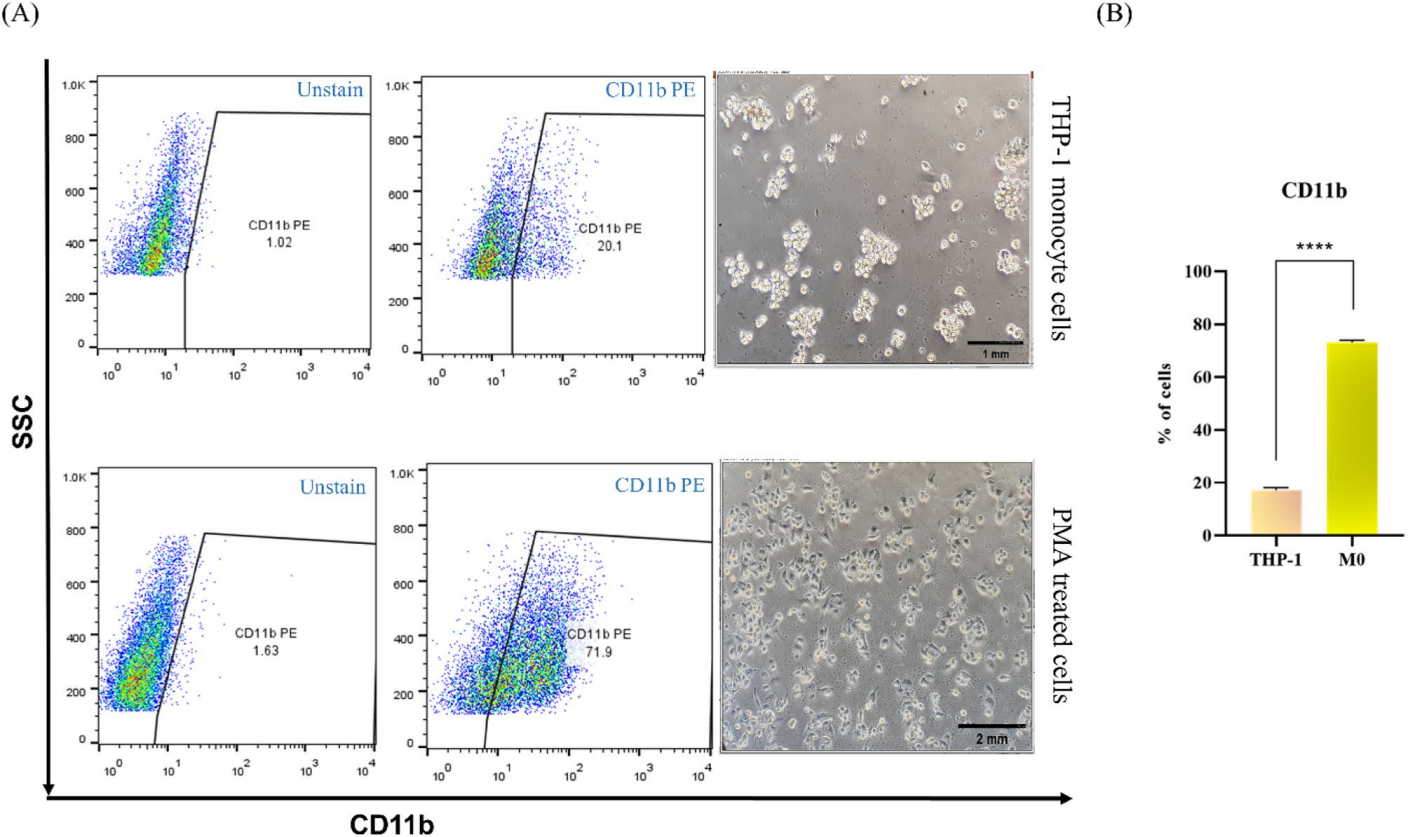
PMA promotes the differentiation of human THP-1 monocytes into macrophages. (**A**) Representative images and flow cytometry analysis of THP-1 Monocytes cells and PMA-treated cells. (**B**) A significant increase in the number of CD11b + cells was detected in the PMA-treated group compared with the THP-1 monocytes cells (*p* < 0.0001). An unpaired t-test was used to make comparisons between groups (Triplicate assays). PMA**,** phorbol myristate acetate.

To investigate macrophage differentiation from monocytes, we measured the expression of CD11b as a differentiation marker in the THP-1 monocytes group and PMA-treated THP-1 cells. An increased expression of CD11b in the PMA-treated group in comparison to the THP-1 monocytes was observed, which suggested that THP-1 monocytes were well-differentiated to MQs by 50 ng/ml PMA for 48 h followed by 24 h rest (Fig. 1).

### Gliadin induced up-regulation of IL-6 cytokine without any effect on TGF-β expression

**Figure 2:**
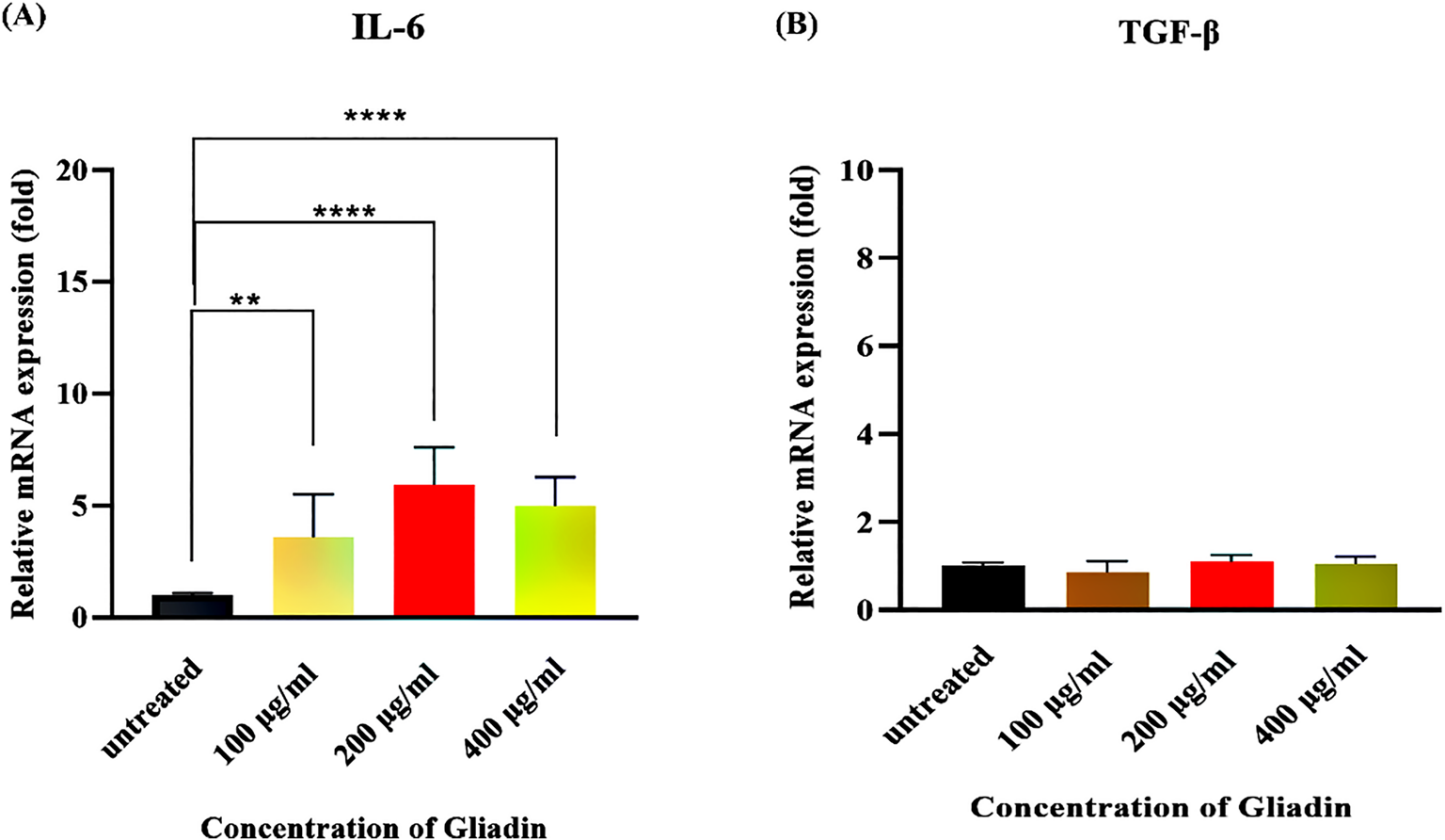
Evaluation of the gliadin effect on THP-1 derived MQs. (**A**) Relative expression of IL-6 was significantly increased in all concentrations of gliadin. (**B**) Relative expression of TGF-β did not reach a statistically significant threshold. (****p < 0.0001, **p < 0.01). The Student’s t-test was used to make comparisons between groups (Triplicate assays).

IL-6 and TGF-β were selected as markers for the immune activity of stimulated MQs. Results showed that the concentration of 200 and 400 μg/ml had the most efficacy in stimulating macrophages to produce IL-6 as an inflammatory cytokine (*p* < 0.0001) (Fig. 2A). In addition, there was no significant difference between various concentrations of gliadin in the expression of TGF-β (*p* = 0.8901) (Fig. 2B).

### *A. muciniphila* can reduce gliadin-induced inflammation in MQs

To characterize the preventive and protective effect of *A. muciniphila,* on gliadin treated MQs, *A. muciniphila* treated MQs (MOI = 1, 10, 100) were treated with gliadin (pre-treat) and gliadin stimulated MQ cells were treated with *A. muciniphila* (MOI = 1, 10, 100) for 24 h (post-treat), respectively. mRNA expressions of IL-6, TGF-β, and IL-10 were assessed by real-time RT-PCR and ELISA methods to evaluate the protein production of TNF-α and IL-10.

**Figure 3:**
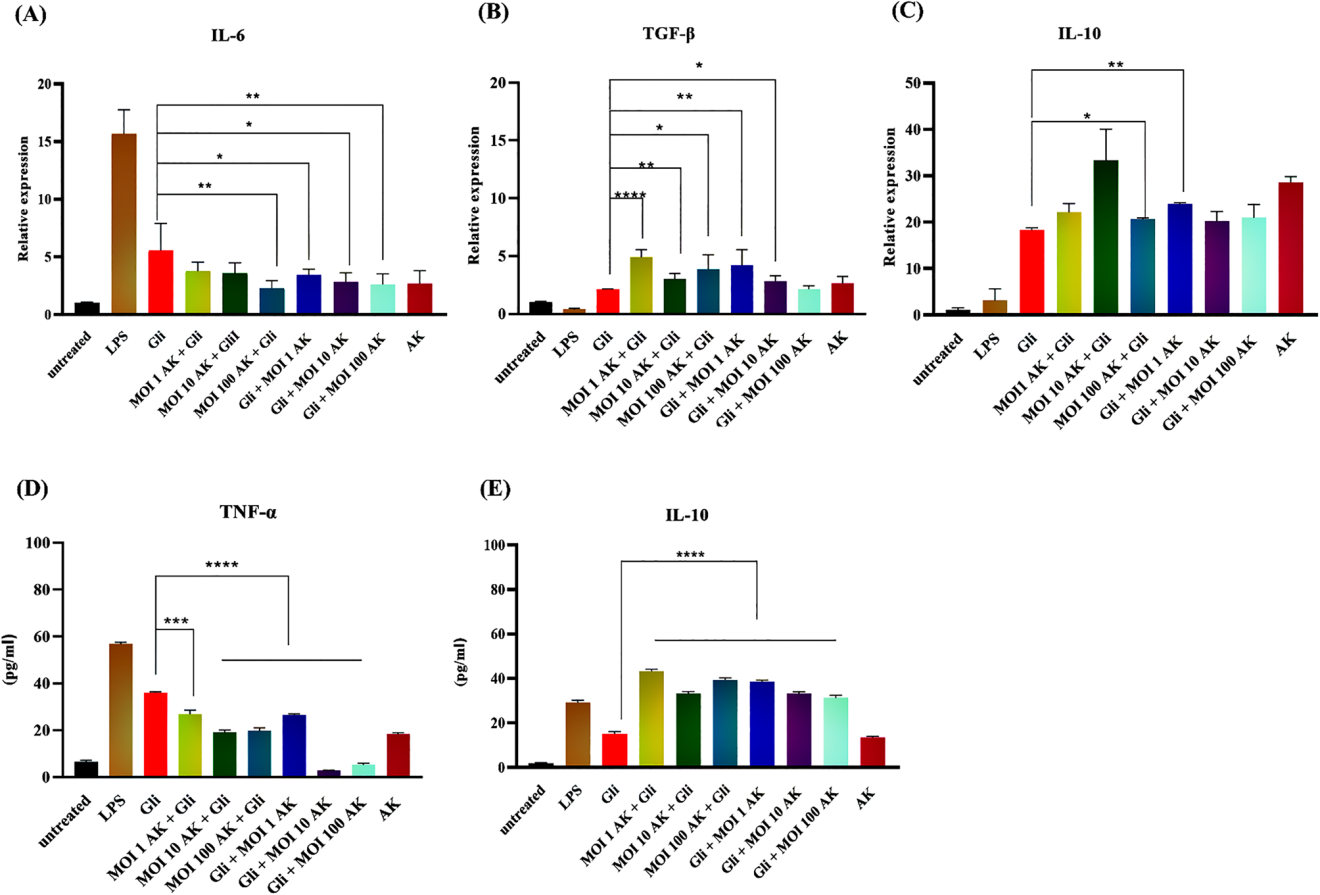
*A. muciniphila* modulates immune-regulatory cytokines*.* (**A**) The qRT-PCR analysis represented a significant reduction of IL-6 expression in the AK (MOI = 100) + Gli group compared to the Gli (gliadin) group. (**B**) Relative expression of TGF-β. (**C**) Relative expression of IL-10. (**D**) TNF-α and (**E**) IL-10 production by MQs with various treatments. Results were analyzed by One-way ANOVA (Triplicate assays) (*p < 0.05, **p < 0.01, ***p < 0.001 and ****p < 0.0001). AK, A. Muciniphila; Gli, gliadin; LPS, lipopolysaccharide; MOI, the multiplicity of infection.

According to the real-time PCR results, pre-treatment of *A. muciniphila* for 24 h significantly reduced IL-6 mRNA expression (MOI = 100) (*p* < 0.01) and increased TGF-β (MOI = 1, 10, 100) (*p* < 0.0001, *p* < 0.01, *p* < 0.05) and IL-10 (MOI = 10) (*p* < 0.01) mRNA expressions relative to the MQ cells stimulated with gliadin. Post-treatment of *A. muciniphila* also significantly decreased IL-6 mRNA levels (MOI = 1,10,100) (*p* < 0.05, *p* < 0.05, *p* < 0.01 respectively) and increased TGF-β (MOI = 1, 10) (*p* < 0.01, *p* < 0.05) mRNA expressions. The changes towards untreated macrophages and LPS stimulated ones were significant in all groups (p˂0.05) (Fig. 3A–C).

As determined by ELISA, pre and post-treatment of gliadin stimulated macrophages with *A. muciniphila* (at all concentrations) could significantly reduce TNF-α and induce IL-10 expressions relative to gliadin-triggered macrophage cells (*p* < 0.0001) (Fig. 3D,E).

### Increased frequency of M2 macrophages in response to *A. muciniphila* treatment

To characterize the effect of gliadin and *A. muciniphila* on the MQs cells phenotype, we assessed the CD80 levels, as an M1-specific marker, and CD206 levels, as an M2 specific marker, by flow cytometry.

**Figure 4:**
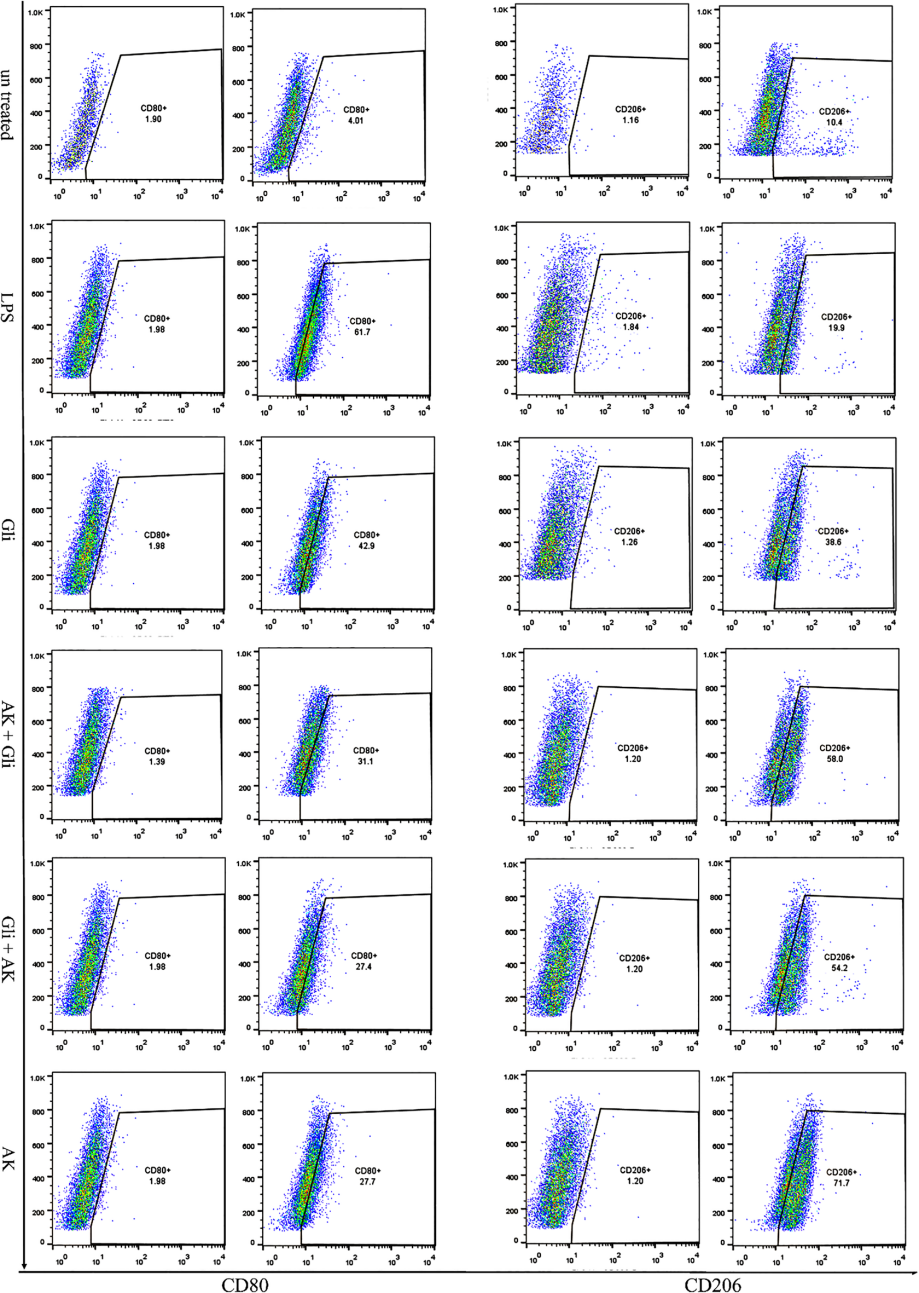
*A. muciniphila* increases the presence of M2 macrophages. Flow cytometry analysis of MQs treated with *A. muciniphila* and gliadin showed a significant decrease in the number of M1 MQ cells compared with the gliadin group. AK + Gli, *A. muciniphila* and gliadin; Gli + AK, gliadin and A. Muciniphila; AK, A. Muciniphila; Gli, gliadin; LPS, lipo poly sacharide. (Duplicate assays) (**p < 0.01, ****p < 0.0001).

Our results showed that incubation of macrophages with gliadin (200 µg/mL) induced a slight increase in the percentage of M1 phenotype compared to the group with no treatment (Fig. 4). Moreover, the results indicated that, pre-and post-treatment of gliadin-stimulated MQs with *A. muciniphila* induced upregulation in the percentage of CD206 expressing cells (M2 phenotype) relative to the group that incubated with gliadin alone (Fig. 4). Furthermore, MQs treatment with *A. muciniphila* increased the percentage of CD206 + cells compared to the untreated group (Fig. 4). The frequency of cells expressing CD80 (M1 phenotype) in the LPS group was increased relative to the untreated cells (Fig. 4).

**Figure 5:**
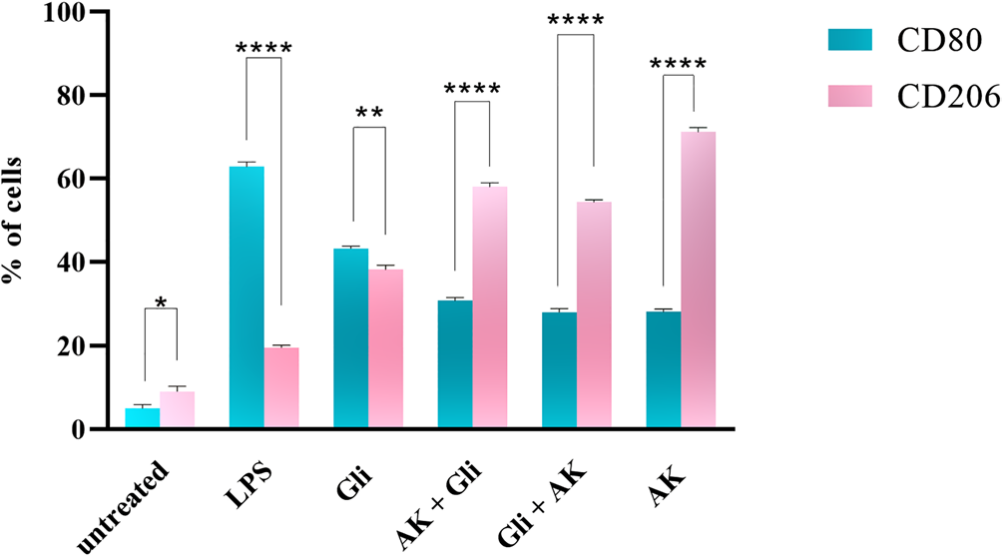
Bar graph chart representing the expression of CD80/CD206 markers in all groups. The student’s t-test was used to make comparisons between markers in each group. AK, A. Muciniphila; Gli, gliadin; LPS, lipo polysaccharide (*p < 0.05, **p < 0.01, ***p < 0.001 and ****p < 0.0001).

A comparison of flow cytometry results in all groups is shown as a bar graph chart (Fig. 5).

## Discussion

Macrophages (MQs) are key players of innate immune response and provide adaptive immunity by acting as antigen-presenting cells (APCs)^18^. These cells can polarize to M1 (pro-inflammatory) or M2 (anti-inflammatory) functional phenotypes in response to their tissue microenvironment^3^. MQs can promote intestinal homeostasis and regulates immune responses and disease progression^19^. Therefore, targeting MQs polarization and inducing M2 phenotype may be a new strategy to treat intestinal inflammatory disorders like celiac disease. In this regard, potential targets that can affect M2 polarization becomes an attractive area of reaching mucosal healing which is the ultimate treatment goal of this kind of disorders^18^. In celiac disease, gliadin peptides induce activation of macrophages toward a pro-inflammatory M1 phenotype^6–10^. In vitro models of gluten toxicity are of particular importance for the development of novel treatment options for CeD. On this basis, in this study we evaluated the inflammatory and anti-inflammatory effects of *A. muciniphila* on THP-1 derived MQs stimulated with gliadin.

Our data showed that, gliadin stimulates pro-inflammatory cytokines (such as TNF-α and IL-6) expression in PMA-stimulated THP-1 MQs, similar to that induced by the potent inflammatory agent LPS and drives their differentiation toward the M1 phenotype. The effect of gliadin on MQ cells has been suggested by numerous in vitro studies conducted on human monocytic cell lines and murine macrophages which show that gliadin elicits an inflammatory response in these cells^6,8,9^. In line with our finding, the study of Serena et al. evaluated the response of primary human monocytes derived MQ to gliadin and indicated that gliadin triggers a potent inflammatory response on human primary MQs^20^.

The positive effects of *A. muciniphila,* on phenotype switching of gliadin triggered M1 MQs to an anti-inflammatory phenotype was also observed in this study. In our model, pre-treatment of gliadin-stimulated MQs with *A. muciniphila* (evaluating the preventive effect) caused increased M2 (CD206 +) phenotype MQ and reduced M1/M2 MQ ratio. This transition from M1 to M2 phenotype was associated with decreased proinflammatory (IL-6, TNF-α) and increased anti-inflammatory (IL-10, TGF-β) cytokines expression^21^.

The ability of *A. muciniphila* to induce an anti-inflammatory environment has been repeatedly reported in the context of different diseases^22,23^. In an in vivo study conducted by Mulhall et al. the effect of *A. muciniphila* and its pili-like protein was evaluated on the MQ’s polarization. In their study, increased alveolar bone loss was induced by *Porphyromonas gingivalis* gavage treatment of mice. The administration of gut commensal microbe *A. muciniphila* or its pili like protein Amuc_1100 to mice orally challenged with *P. gingivalis*, could protect alveolar bone loss and direct differentiation of MQs toward M2 phenotype and also increase IL-10 gene and protein expression within the gingival tissue^24^. Keshavarz Azizi Raftar et al. in their recent in vivo study evaluated the effects of live and pasteurized *A. muciniphila* and its extra cellular vesicles (EVs) on inflammatory markers involved in liver fibrosis. According to their results, Live *A. muciniphila* and its EVs had inhibitory effects on liver inflammation and hepatocytes damages. Moreover, four weeks of oral gavage with live and pasteurized *A. muciniphila* and its EVs could successfully maintain homeostasis in the colon tissue and enhances the epithelium and mucosal layer thickness and crypt depth accompanied by an increase in RNA level of tight junction proteins^25^. Ashrafian et al. also in their study showed that alive and pasteurized *A. muciniphila* had positive effect on tight junction and immune response-related genes in Caco-2 cell line^26^.

It is worth mentioning that *A. muciniphila* has mucin-degrading enzymes and utilizes mucin, a complex glycosylated protein, and degrades it as carbon, energy and nitrogen sources^27^. These beneficial by-products are involved in regulating the host immune system through different signals including TNF‐α, IL‐10, etc.^14,28^.

In the aspect of the protective effect of *A. muciniphila,* our results also showed that treating gliadin stimulated MQs with *A. muciniphila* leads to anti-inflammatory responses too*.* In general, no significant difference was observed between the protective and preventive effects of *A. muciniphila.*

This is also noteworthy that, different probiotics like some strains of *Bifidobacterium* have modulatory effects on immune responses through using different strategies, like reducing IL-6 levels^29^.

On the other hand, *A. muciniphila* alone also induced the increased levels of IL-6 and TNF-alpha in THP-1 cells, which may indicate its pivotal effect on the modulation of immune responses and point to the need for further investigation for finding its precise mechanisms. Actually, it can be said that, the best anti-inflammatory effect of *A. muciniphila* is achieved when it is used following inflammatory conditions.

Here, we gathered current suggestions regarding the anti-inflammatory and immune-modulatory mechanisms of *A. muciniphila*. As mentioned above, one observed mechanism is that purified Amuc_1100, as an outer-membrane protein of *A. muciniphila*, can activate TLR2 signaling which is associated with the production of specific cytokines and mainly leads to high levels of IL-10. Amuc_1100 is showed to be effective in reducing the inflammatory state of the immune responses, improving the barrier integrity and maintaining host immunological homeostasis^16^. Moreover, short-chain fatty acids (SCFAs) and branched-chain fatty acids (BCFAs) are the main metabolites of *A.* *muciniphila*, that may have a role in alleviating the inflammatory responses^14,30^. In general, SCFAs are reported to play a role in regulating the immune system and inflammatory responses and BCFAs are considered to have a beneficial role against inflammation and affect the expression of anti-inflammatory cytokines^31–33^. *A.* *muciniphila*-derived EVs have also been reported to have immune-modulatory and anti- inflammatory properties^34^. In this regard, the significant effects of EVs on the modulation of inflammatory and anti-inflammatory mediator’s gene expression, lipid metabolism and intestinal homeostasis has been reported in previous studies on different pathological conditions^25,34^. Additionally, it has been shown that, *A. muciniphila* adheres to the intestinal epithelium and increases the integrity of the epithelial cell layer. This observation suggests the ability of *A. muciniphila* to strengthen an impaired gut barrier and its linkage with intestinal health^35^.

The main limitation of this study was that we could not fully explain the exact beneficial mechanism of *A. muciniphila* on gliadin stimulated THP-1- derived MQ cells and further studies are highly recommended to expand our knowledge in this regard. Moreover, due to the significant difference between the in vitro and in vivo systems, the results should be used with caution and more extensive and detailed research is needed to confirm our results. Establishing a celiac disease animal model to prove these in vitro findings is also encouraged. Moreover, bone marrow-derived macrophages can be used to prove the equal anti-inflammatory effect of *A. muciniphila *ex vivo.

As celiac disease is accompanied by oral and intestinal dysbiosis, it is recommended that finding novel supplementary therapeutic targets with the aim of reducing dysbiosis harmful effects, can improve CeD patient’s quality of life. This study suggests that, *A. muciniphila* as a beneficial bacterium have the ability to change MQs response to gliadin and induce anti-inflammatory responses and may be considered as a supplemental therapy for CeD patients. The results might be expanded to next pre-clinical studies.

## Methods

### Cell culture

Human monocytic THP-1 cell line was purchased from Pasteur Institute of Iran. The cells were cultured in RPMI 1640 (Biosera, France) medium supplemented with 10% (v/v) fetal bovine serum (FBS) (Gibco, USA) and a 1% (v/v) mixture of penicillin and streptomycin (Gibco, USA). cells were maintained in a humidified incubator containing 5% CO_2_ at 37 °C. The culture medium was changed every 48 h.

### Macrophage’s differentiation

For macrophage differentiation, 1× ${10}^{6}$ cells THP-1 was seeded in each well of 6-well tissue culture plates (SPL Life Sciences, Korea) and treated with 50 ng/ml phorbol 12-myristate 13-acetate (PMA; Santa Cruz Biotechnology Cat. No. sc-3576) for 36 h. To prove the differentiation of monocytes to macrophages, in addition to examining the morphology of cells, the CD11b expression assay was performed by flow cytometry technique^36^.

### Bacterial culture

*A. muciniphila MucT* (ATCC BAA-835) was grown anaerobically (N2/CO_2_ 80:20 v/v) in brain heart infusion broth supplemented with 3% commercial mucin (Sigma -Aldrich) in 10 ml Hungate anaerobic tubes. The concentration of bacteria was determined by measuring the optical density at 600 nm. After the OD600 reached ~ 1, bacterial suspension was centrifuged at 11,000 g for 20 min. Liquid cultures were washed and resuspended in anaerobic sterile PBS at the required concentration prior to each experiment^34,37^.

### Gliadin and bacteria treatments

Gliadin (Sigma, USA) was dissolved in 1% Dimethyl sulfoxide (DMSO). In order to determine the appropriate concentration of gliadin with the most inflammatory effect on MQs, three different concentrations of gliadin (100, 200, 400 μg/ml) were used (although previous studies considered the concentration of 200 μg/ml as the best concentration for gliadin in this regard)^6^. Based on our findings, the concentration of 200 μg/ml of gliadin was used as the optimal concentration too.

*A. muciniphila* were added to MQ’s culture at a multiplicity of infection (MOI) of 1, 10 and 100 respectively^25^.

To investigate the effect of *A. muciniphila* on the MQ’s response to gliadin peptide, *A. muciniphila* was added to the MQ cells before stimulation with gliadin as pre-treat (preventive effect) and after stimulation with gliadin as post-treat (protective effect).

**Figure 6:**
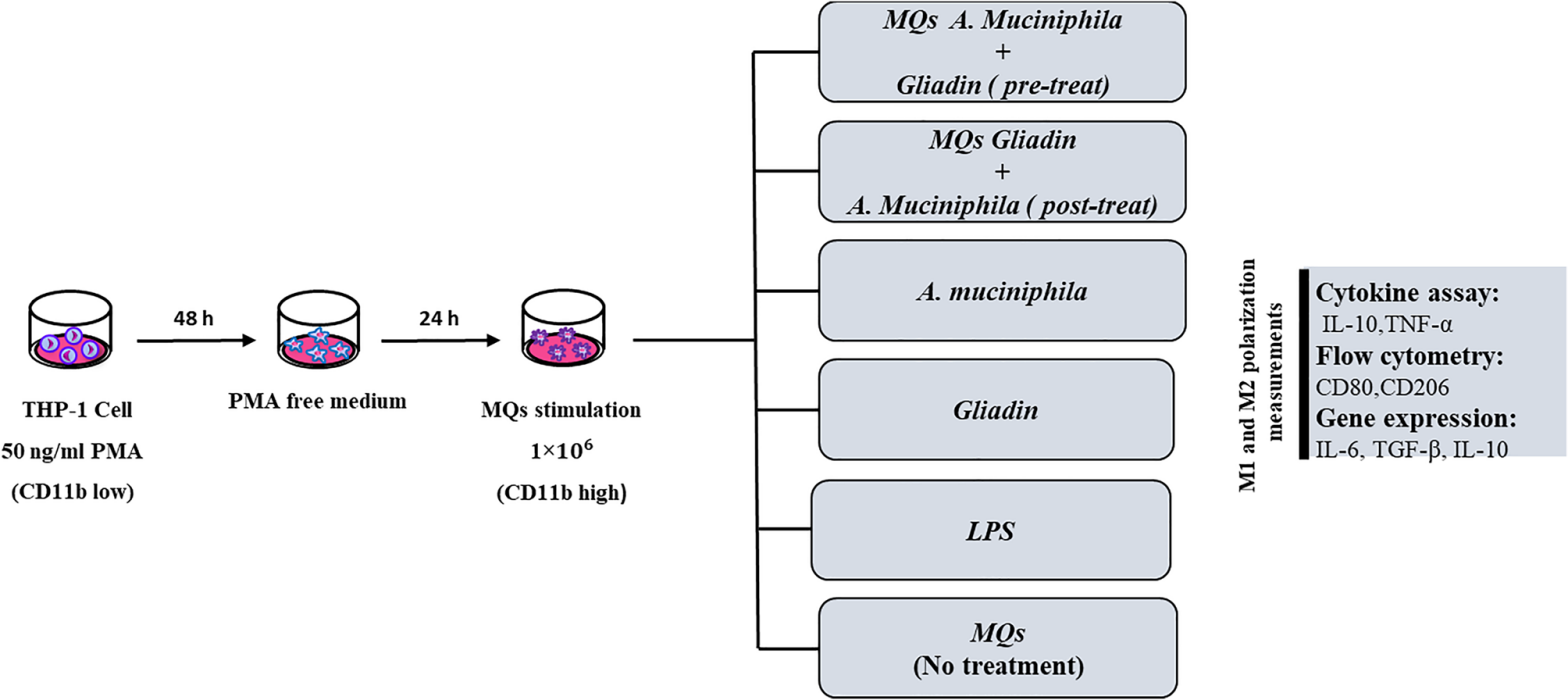
Schematic illustration of the study design.

To evaluate the preventive effect of *A. muciniphila* on the inflammatory effect of gliadin, 1 × 10^6^ of THP-1 cells were seeded in 6 well cell culture treated plate. After 48 h of PMA treatment, the culture media with PMA was changed with penicillin and streptomycin-free medium (to prevent bacterial death) followed by rest for 24 h prior to experiments. Cells were treated with *A. muciniphila* in three concentrations (MOI = 1, 10, 100) for 24 h and then the treatment with gliadin was done for the next 24 h (pre-treat). Furthermore, to evaluate the protective effect of *A.muciniphila*, after 24 h stimulation with gliadin, cells were treated with different concentrations (MOI = 1, 10, 100) of *A. muciniphila* (post-treat). A well was treated with 20 ng/ml LPS (Santa Cruz Biotechnology Cat No. sc-3535), as positive control, to compare its induction pattern with gliadin and bacteria. Two wells that were treated with gliadin alone and *A. muciniphila* alone and a well full of PMA-treated THP-1 cells without any treatment was considered as the negative control group (Fig. 6). Experiments were performed in triplicate (technical replicates).

### RNA extraction and quantitative real -time PCR (qRT -PCR)

Total RNA was extracted from THP-1 monocytes derived macrophage cultures using the Total RNA Purification Mini kit for Blood/Cultured Cell/Tissue (Yekta Tajhiz Azma, Tehran, Iran) according to the provided protocol. Reverse transcription was performed using 2-step 2X RT-PCR Premix (Taq) kit (BioFact™, South Korea), according to the manufacturer's instructions. Quantitative RT-PCR was conducted on the thermocycler Rotor Gene Q MDx (QIAGEN) with SYBR Green Master Mix (BioFact™, South Korea). Glyceraldehyde 3-phosphate dehydrogenase (GAPDH) was measured as a housekeeping reference gene in each qPCR run and relative quantitation (RQ) for each gene expression was calculated by 2^−ΔΔCt^ Method (ΔCt = Ct_target_ – Ct_house keeping_). Specific human primers for IL-6, IL-10, and TGF-β were designed and analyzed by Gene Runner v. 3.05 software and used to evaluate gene expression (Table 1). All samples were assayed in triplicate.

**Table 1:** Sequences for forward and reverse primers used to measure genes expression by real-time RT-PCR.

Gene symbol	Primer sequence	Reference
IL-6	F: 5′-CTGGATTCAATGAGGAGACTTGC-3′ R: 5′-TCAAATCTGTTCTGGAGGTACTCTAGG-3′	Tian et al.^38^
TGF-β	F: 5′-CAATTCCTGGCGATACCTCAG-3′ R: 5′-GCACAACTCCGGTGACATCAA-3′	Jin et al.^39^
IL-10	F: 5′-AAGAAGGCATGCACAGCTCA-3′ R: 5′-AAGTGGGTGCAGCTGTTCTC-3′	Aghamohamadi et al.^40^
GAPDH	F: 5′-TCTGACTTCAACAGCGACAC-3′ R: 5′-TACTCCTTGAGGCCATGT-3′	This study

### ELISA

Following stimulations, culture supernatants were harvested and stored at ˗70 °C until use. The level of IL-10 and TNF-α was determined using sandwich enzyme-linked immunosorbent assay (ELISA) kits (KPG. Kerman. Iran) according to the manufacturer’s instructions. Triplicate results were read by an ELISA reader at 450 nm (TECAN, Salzburg, Austria).

### Flow cytometry

PE-conjugated anti-CD11b (BioLegend, San Diego, USA) was used as a difference marker between THP-1 cell line monocytes and differentiated MQs. MQs were stained with FITC-conjugated anti-CD80 (BioLegend, San Diego, USA) and PerCP-eFlour710 conjugated anti-CD206 (BD bioscience, USA) antibodies to evaluate the percentages of M1 and M2 MQs, respectively. The duplicate data were analyzed by FlowJo software v10 (FlowJo LCC, Ashland, OR, USA).

### Statistics

Statistical analysis was performed using GraphPad Prism v8 (GraphPad Software, San Diego, CA, USA). Two groups were compared by Student's t-test, and One-way ANOVA was applied for comparisons between multiple experimental groups. *p* values were considered significant at less than 0.05. The criteria for significance were *p < 0.05, **p < 0.01, ***p < 0,0.001 and ****p < 0.0001.

## Data Availability

The datasets generated during and/or analysed during the current study are available from the corresponding author on reasonable request.
